# Extrachromosomal circular DNA (eccDNA) characteristics in the bile and plasma of advanced perihilar cholangiocarcinoma patients and the construction of an eccDNA-related gene prognosis model

**DOI:** 10.3389/fcell.2024.1379435

**Published:** 2024-06-06

**Authors:** Shijie Fu, Yuyang Dai, Pengjun Zhang, Kanglian Zheng, Guang Cao, Liang Xu, Yujie Zhong, Chuanxin Niu, Xiaodong Wang

**Affiliations:** Key Laboratory of Carcinogenesis and Translational Research (Ministry of Education/Beijing), Department of Interventional Oncology, Peking University Cancer Hospital and Institute, Beijing, China

**Keywords:** perihilar cholangiocarcinoma, extrachromosomal DNAs, hepatic arterial infusion chemotherapy, immune response, prognosis

## Abstract

Extrachromosomal DNAs (eccDNAs) frequently carry amplified oncogenes. This investigation aimed to examine the occurrence and role of eccDNAs in individuals diagnosed with advanced perihilar cholangiocarcinoma (pCCA) who exhibited distinct prognostic outcomes. Five patients with poor survival outcomes and five with better outcomes were selected among patients who received first-line hepatic arterial infusion chemotherapy from June 2021 to June 2022. The extracted eccDNAs were amplified for high-throughput sequencing. Genes associated with the differentially expressed eccDNAs were analyzed using Gene Ontology (GO) and Kyoto Encyclopedia of Genes and Genomes (KEGG) pathway analyses. The differentially expressed bile eccDNA-related genes were used to construct a prognostic model. Across all 10 patients, a total of 19,024 and 3,048 eccDNAs were identified in bile and plasma, respectively. The concentration of eccDNA detected in the bile was 9-fold higher than that in plasma. The chromosome distribution of the eccDNAs were similar between bile and matched plasma. GO and KEGG pathway analyses showed enrichment in the mitogen-activated protein kinase (MAPK) and Wnt/β-catenin pathways in patients with poor survival outcomes. According to the prognostic model constructed by eccDNA-related genes, the high-risk group of cholangiocarcinoma patients displayed significantly shorter overall survival (*p* < 0.001). Moreover, the degree of infiltration of immunosuppressive cells was higher in patients in the high-risk group. In conclusion, EccDNA could be detected in bile and plasma of pCCA patients, with a higher concentration. A prognostic model based on eccDNA-related genes showed the potential to predict the survival and immune microenvironment of patients with cholangiocarcinoma.

## 1 Introduction

1. Based on the anatomical location, cholangiocarcinoma (CCA) is typically classified into intrahepatic CCA, perihilar CCA (pCCA), and distal CCA (Rizvi et al., 2018). Approximately half of all cholangiocarcinoma (CCA) cases are attributed to pCCA, predominantly located in the perihilar bile duct (Banales et al., 2016). Gemcitabine and cisplatin (CisGem) remain the first-line systemic chemotherapy options for advanced pCCA, with a 5-year overall survival (OS) rate of approximately 5% and a median overall survival duration of less than a year (Palmieri et al., 2020). We have demonstrated the safety and efficacy of hepatic arterial infusion chemotherapy (HAIC) with oxaliplatin and 5-fluorouracil in a prospective phase II trial, with a median progression-free survival (PFS) duration of 12.2 months and a median overall survival (OS) duration of 20.5 months (Wang et al., 2017). However, the survival benefits of HAIC for pCCA vary. At present, the molecular mechanisms governing the differential prognoses observed in pCCA patients who undergo HAIC are incompletely understood.
2. Extrachromosomal DNAs (eccDNAs) have a closed circular structure and are separate from the 22 human linear autosome pairs and pair of sex-determining chromosomes (Shibata et al., 2012). Initially described as double minutes, eccDNAs could be detected in children with malignant tumors over half a century ago (Cox et al., 1965). Next-generation sequencing studies have revealed that eccDNAs exist in healthy human somatic tissues (Møller et al., 2018a) and maternal and fetal plasma (Sin et al., 2020). In cancers, eccDNAs frequently carry oncogenes, leading to genetic heterogeneity and promoting tumor progression (Turner et al., 2017). EccDNAs can participate in oncogenesis by driving oncogenic genome remodeling in neuroblastoma (Koche et al., 2020). In addition, oncogenic eccDNAs can operate as mobile enhancers, thereby increasing chromosomal transcription to support the progression of tumors (Zhu et al., 2021). Sequencing data obtained from a substantial population of cancer patients has demonstrated that eccDNAs correlate with the amplification of oncogenes in various cancer types (Kim et al., 2020). However, the presence and function of eccDNAs remain unknown in advanced pCCA. Recent cancer research on eccDNAs has mainly centered on tumor cell lines, tissue samples and blood samples. Knowledge of eccDNAs in bile samples remains limited. This study investigated eccDNAs in bile and plasma samples obtained from patients with advanced pCCA and the potential value of eccDNA as a survival biomarker in pCCA patients receiving HAIC.

## 2 Materials and methods

### 2.1 Subjects

This study was approved by the institutional review board of Peking University Cancer Hospital (approval protocol number: 2021KT144). A retrospective review of the Hospital Information System identified 31 advanced pCCA patients who received first-line HAIC treatment from June 2021 to June 2022 at our center. Bile or plasma samples were obtained from 18 patients prior to treatment. According to complete survival follow-up data, all patients died, with a survival distribution as follows: OS < 6 months (n = 7), 6 months < OS < 12 months (n = 4), and OS > 12 months (n = 7). We randomly selected five patients among seven with a better prognosis (OS > 12 months; Group A) and five patients among seven with a poor prognosis (OS < 6 months; Group B) from the biobank for this study.

### 2.2 Data collection and processing

The Cancer Genome Atlas Program (TCGA) database (https://portal.gdc.cancer.gov/) was used to download the cholangiocarcinoma (TCGA-CHOL) RNA-seq dataset (Chang et al., 2013) from the Gene Expression Omnibus (GEO) database (https://www.ncbi.nlm.nih.gov/geo/) to obtain the GSE107943 cholangiocarcinoma dataset (Edgar, Domrachev, and Lash, 2002). The cholangiocarcinoma E-MTAB-6389 dataset was obtained from ArrayExpress (https://www.ebi.ac.uk/biostudies/arrayexpress) (Sarkans et al., 2021). The gene expression values of the TCGA-CHOL and GSE107943 datasets were then normalized to log_2_ (TPM+1).After sorting the dataset and excluding the data with incomplete prognostic information and normal sample data, the merged cohort (n = 142) was created by combining the TCGA-CHOL (n = 36), E-MTAB-6389 (n = 76), and GSE107943 (n = 30) dataset cohorts using the R package “inSilicoMerging”. Furthermore, batch effects were removed using the R package “combat” (Supplementary Material S6).

### 2.3 Treatment and follow-up

HAIC was performed as previously described (Hu et al., 2019). The HAIC treatment, referred to as “3cir-OFF,” consisted of administering oxaliplatin (40 mg/m2 for 2 h), 5-fluorouracil (800 mg/m2 for 22 h), and intravenous folinic acid (200 mg/m2) over a period of three consecutive days, repeated every three or 4 weeks. Response Evaluation Criteria in Solid Tumors (RECIST) (version 1.1) were utilized to assess treatment response. Overall survival (OS) was calculated from the initiation of HAIC until death. Progression-free survival (PFS) was determined from the start of HAIC until either tumor progression or death, whichever occurred first.

### 2.4 Bile and blood samples

Bile samples (15 mL) were obtained by percutaneous transhepatic cholangial drainage (PTCD). Peripheral blood samples (10 mL) were obtained by venipuncture within 7 days before the first cycle of the HAIC procedure and collected into anticoagulant tubes. The fresh bile and peripheral blood samples were centrifuged at 2,000 rpm for 10 min; next, the supernatant was stored in a refrigerator at −80°C. All patients signed informed consent documentation for clinical specimen collection before PTCD and HAIC treatment.

### 2.5 DNA preparation and eccDNA sequencing

CloudSeq Biotech Inc. (Shanghai, China) sequenced eccDNA according to previously established experimental procedures (Møller et al., 2018a). DNA extraction from either bile or plasma samples was performed using the QIAamp Circulating Nucleic Acid Kit (QIAGEN Sciences, Inc., Germantown, MD, USA). Subsequently, 25 ng of bile or plasma DNA was treated with 1 μL of Plasmid-Safe ATP-dependent DNase (Epicenter, Madison, WI, USA) within a 50-μL reaction system and maintained at 37°C for 5 min. Afterward, column purification was performed using the MinElute Reaction Cleanup Kit (QIAGEN Sciences, Inc.) to remove any linear DNA. EccDNA isolated from the bile or plasma samples was enriched using the Nextera XT DNA Library Preparation Kit (Illumina, Inc., San Diego, CA, USA). The resulting DNA libraries were then sequenced using an Illumina NovaSeq 6000 platform.

### 2.6 Sequencing analysis of eccDNA

Q30 quality control was conducted on the paired-end reads. Subsequently, circle-map software (v1.1.4) was used to identify eccDNA, while samtools (v0.2) was used to retrieve the raw soft-clipped read counts at the breakpoint. EdgeR was used to conduct between-samples statistical analyses, counts per million (CPM) was used to standardize the detected split read, and the subsequent difference analysis was conducted according to standardized data (Robinson et al., 2010). Differential analysis was conducted using DESeq2 on standardized data, and the differential genes in the group were screened according to the calculated fold change values (*p* < 0.05, fold change >2.0). Genes linked to these differentially expressed eccDNAs were used to perform GO and KEGG pathway enrichment analyses.

### 2.7 Kaplan‒Meier (K–M) curve plotting

The “survival” and “survminer” R packages were utilized to generate K‒M curves to illustrate the disparity in prognosis among distinct cohorts of patients categorized as high-risk and low-risk.

### 2.8 ROC curve plotting

ROC curves were generated using the R packages “survival,” “survminer,” and “timeROC” to assess the model’s predictive ability for 1-, 3-, and 5-year survival.

### 2.9 Analysis of immune characteristics

The analysis of enrichment scores for 29 immune-related gene sets in each sample was performed using the R packages “GSEABase” and “GSVA”. Subsequently, the “limma” and “ggpubr” packages were used to identify and illustrate the immune gene sets that differed between the high- and low-risk groups.

### 2.10 Function enrichment analysis

Gene set enrichment analysis (GSEA) was conducted using version 3.0 of the GSEA software obtained from the GSEA website. Relevant pathways and molecular mechanisms were assessed by acquiring gene sets from the Molecular Signatures database. The gene set size ranged from 5 to 5000, with 1,000 resampling iterations. A *p*-value <0.05 and FDR <0.25 were considered to indicate significance. GSVA analysis was performed using the R packages “GSEABase” and “GSVA” to calculate the enrichment score for each gene per sample to obtain the enrichment score matrix. Further analysis and visualization of the gene sets that displayed differences between the high- and low-risk groups were conducted using the “limma” and “pheatmap” packages.

### 2.11 Statistical analysis

Differently expressed eccDNAs between two groups were identified by *t*-test. A *p*-value <0.05 was considered to indicate significance. All of the analyses were performed using SPSS v.23.0 software (IBM Corp, Armonk, NY, USA).

## 3 Result

### 3.1 Patient characteristics

Table 1 presents the baseline traits of the individuals and their PFS and OS outcomes. Notably, patients in group A exhibited a considerably longer OS than those in group B [25 months (95% CI: 22.8–27.2 months) vs. 3 months (95% CI: 1.9–4.1 months), *p* = 0.002]. The PFS of patients in group A was also longer than that of patients in group B [19 months (95% CI: 4–34 months) vs. 1.9 months (95% CI: 1.7–2.1 months), *p* = 0.002].

**TABLE 1:** Summary of patient baseline characteristics.

Patient	P1	P2	P3	P4	P5	P6	P7	P8	P9	P10
Age (Y)	56	68	34	53	42	92	58	70	54	73
Sex	Female	Male	Female	Female	Male	Male	Male	Male	Male	Male
HBV infection	None	Yes	None	None	None	None	None	Yes	None	None
Child-Pugh class	B	B	B	B	B	A	B	A	B	B
CEA (ng/mL)	2.45	4.06	1.23	1.93	1.04	4.42	5.44	1.99	2.04	6.68
CA19-9 (U/mL)	1978	2,614	2003	330	1,508	94.94	246.3	1,080	11.29	7,401
Total bilirubin (μmol/L)	93.6	100	97.75	134.1	68.8	31.9	78.4	74	108.4	147.4
ECOG performance status	0	0	1	0	0	0	1	1	0	1
Extent of disease	N0M0	N1M0	N2M1	N0M0	N1M0	N0M0	N0M0	N0M0	N0M0	N0M0
HAIC cycles	2	2	2	2	2	4	6	6	6	6
PFS (months)	1.6	2.2	1.9	2.0	1.8	10.0	12.0	19.0	25.0	6.0+*
OS (months)	5.5	3.0	2.5	6.0	2.0	25.0	14.0	25.0	30.5	22.5
Bile sample	Yes	Yes	Yes	Yes	None	Yes	Yes	Yes	Yes	Yes
Plasma sample	Yes	None	None	Yes	Yes	Yes	None	Yes	Yes	Yes
Treatment response	PD	PD	PD	PD	PD	PR	PR	SD	PR	PR
Group	B	B	B	B	B	A	A	A	A	A

*: Based on the date of last imaging, the PFS, of P10 was recorded as 6+ months.

CEA: carcinoembryonic antigen; CA19-9: carbohydrate antigen 19-9; HAIC: hepatic arterial infusion chemotherapy; PFS: progression-free survival; OS: overall survival; PR: partial response; SD: stable disease; PD: progressive disease; HBV: hepatitis B virus; ECOG, eastern cooperative oncology group.

### 3.2 Genome-wide detection of eccDNAs in bile and plasma samples

**FIGURE 1:**
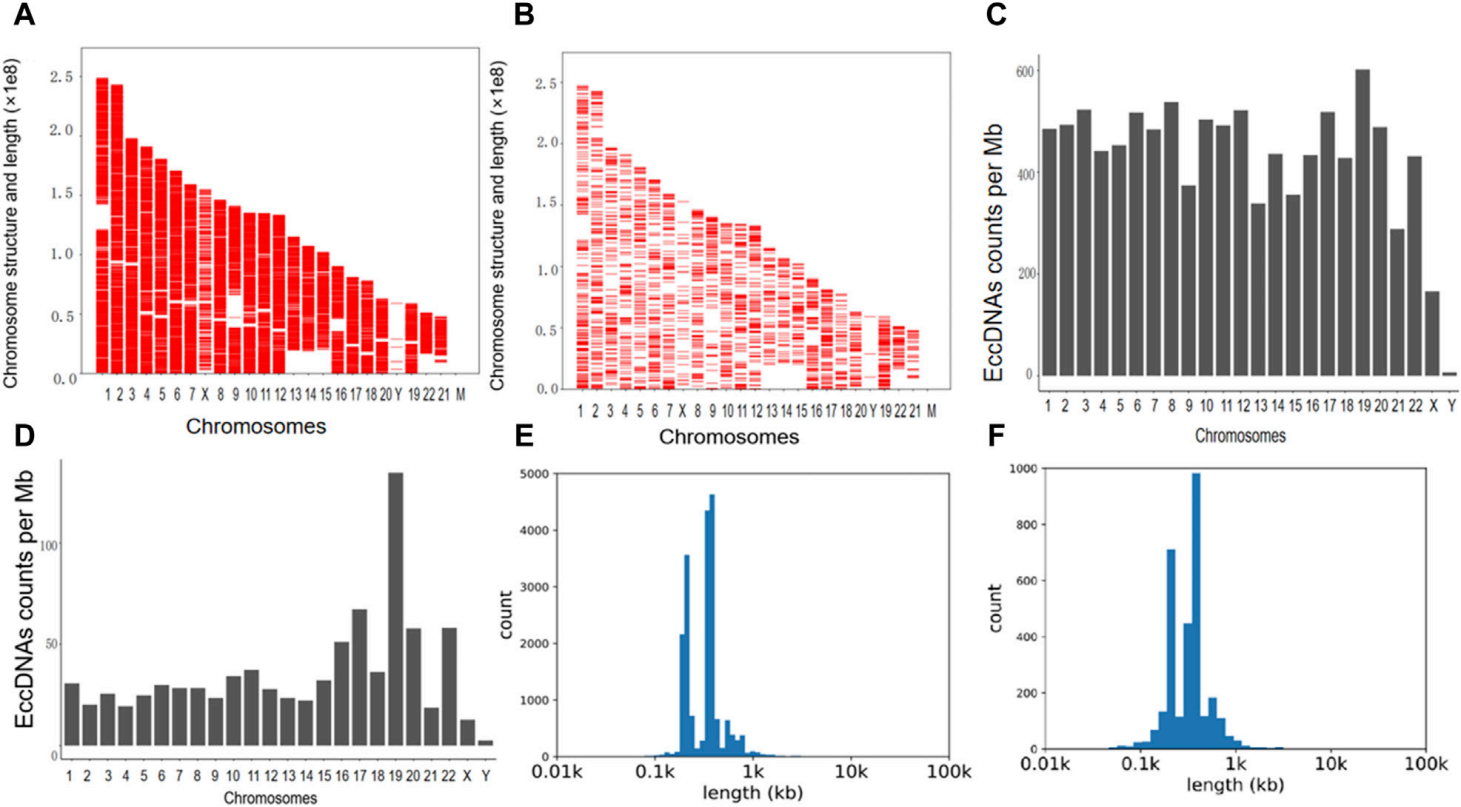
The presence and length distribution of eccDNA in bile and plasma. **(A,B)** The presence of eccDNAs in the bile and corresponding plasma samples is illustrated in the Venn diagram. **(C,D)** The frequency of eccDNAs in bile and plasma samples. **(E,F)** Length distribution of eccDNA at ≤ 100,000 bp in bile and plasma. eccDNAs, extrachromosomal circular DNAs.

The cleaned reads obtained through high-throughput sequencing were aligned with the human genome (UCSC hg19) to identify eccDNAs in the bile and plasma samples. In total, 19,024 eccDNAs were annotated to the 23 pairs of chromosomes in bile samples, while seven plasma samples contained 3,048 eccDNAs. Analysis of the genomic distribution of eccDNAs revealed their presence in all 23 pairs of chromosomes, as shown in Figures 1A, B. Notably, eccDNAs originating from mitochondria were excluded before high-throughput sequencing and, hence, were not detected. The frequency of eccDNAs per Mb was relatively consistent across each chromosome in both bile and plasma samples, as depicted in Figures 1C, D. Notably, the length distribution of eccDNAs in bile samples ranged from 38 to 5,351,028 bp, with a prominent peak observed at approximately 550 bp (Figure 1E). Similarly, plasma samples exhibited a length distribution ranging from 38 to 883,052 bp, with a peak also observed at 550 bp (Figure 1F). These findings demonstrate that eccDNAs are prevalent in both bile and plasma samples from patients with pCCA.

### 3.3 Comparison of eccDNA distribution patterns between the bile and matched plasma samples

**FIGURE 2:**
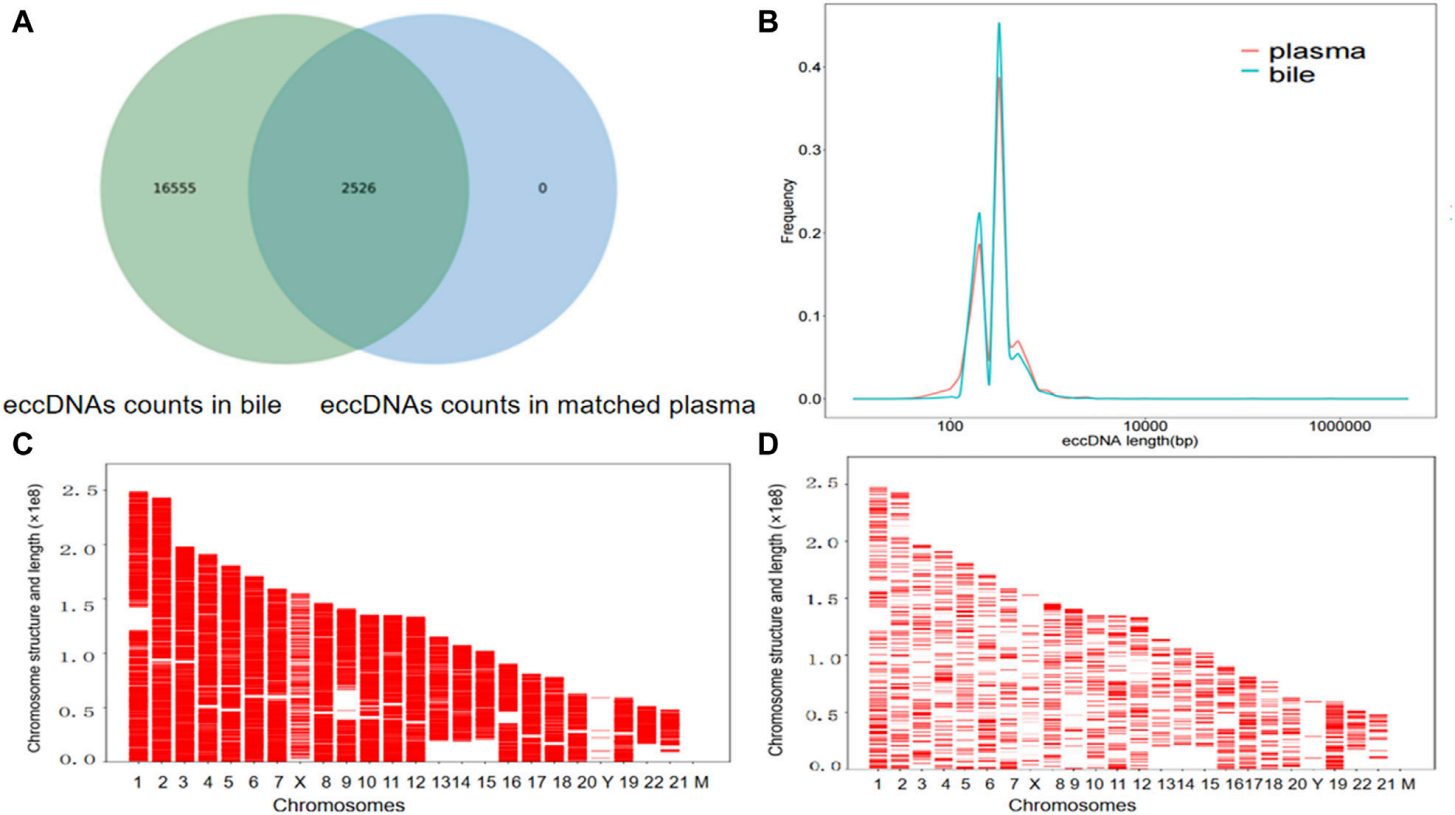
The presence of eccDNAs in bile and matched plasma samples. **(A)** Venn diagram showing the eccDNAs in the bile and matched plasma samples. **(B)** Length distribution of eccDNAs in the bile and matched plasma samples. **(C)** Distribution of eccDNAs in the 23 pairs of chromosomes in six bile samples. **(D)** A comparative examination of the distribution of eccDNAs across the 23 pairs of chromosomes in the six plasma samples corresponding to the bile samples.

For the six patients (patients 1, 4, 6, 8, 9, and 10) with both bile and matched plasma samples, overlap comparisons showed that out of 19,081 eccDNAs, 16,555 eccDNAs were exclusively observed in bile samples, 2,625 were identified in both bile and plasma samples, and no eccDNAs were exclusively detected in plasma samples; the level of eccDNA in the bile was 9-fold higher than in the plasma (Figure 2A). In the group comparison of these six individuals, similar characteristics were observed in the length distribution of bile samples and corresponding plasma samples, including features such as the peak location and length range (Figure 2B). The length distribution for each matched sample also showed similar peak locations (Supplementary Material S1). In addition, the chromosome distribution of the eccDNAs was similar between the bile and matched plasma in these six patients (Figures 2C, D).

### 3.4 Investigation into the disparity in eccDNA expression in the bile between group A and group B

**FIGURE 3:**
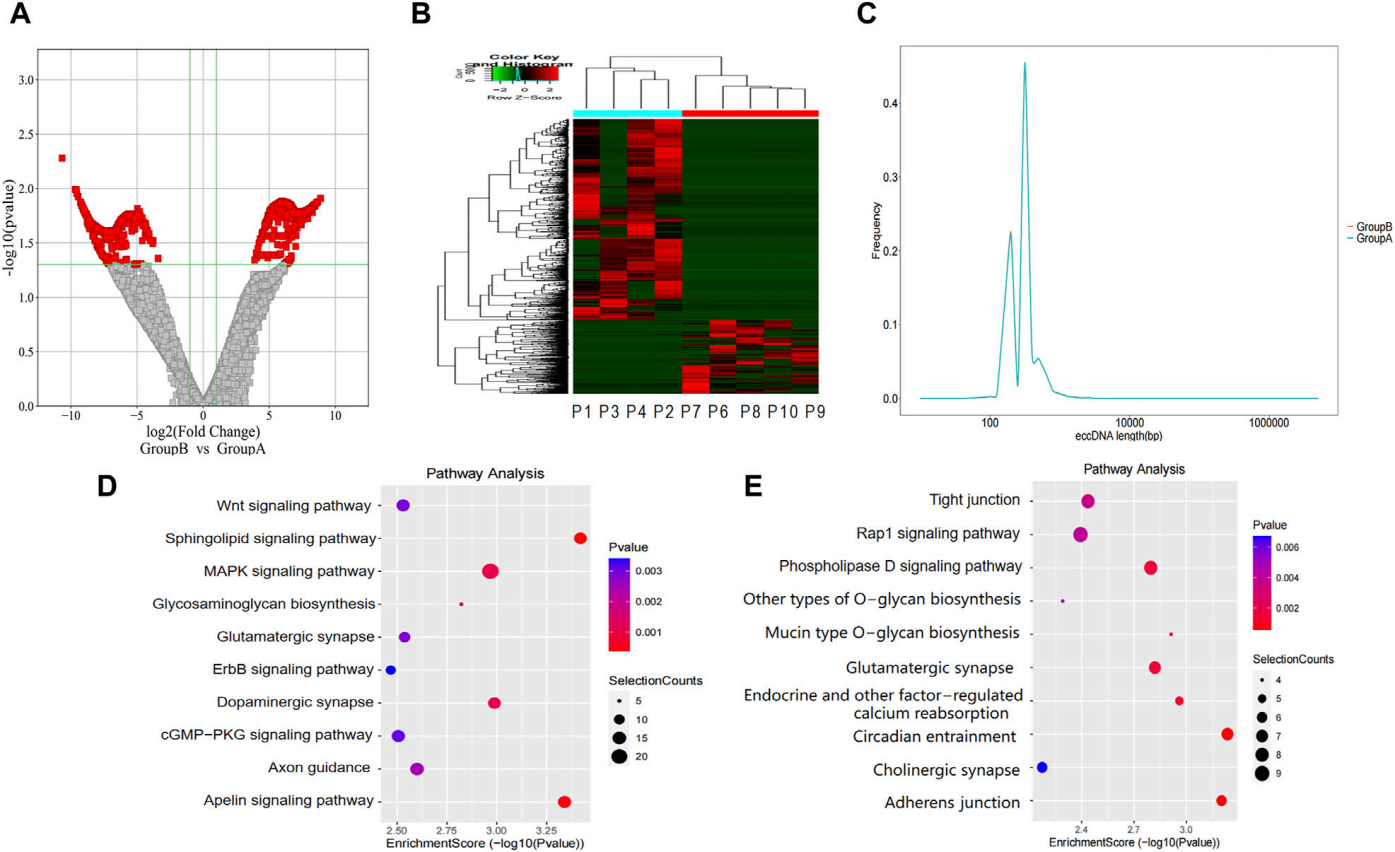
Differentially expressed eccDNAs in bile between group A and group B. **(A,B)** Scatter and cluster plots showing the differentially expressed eccDNAs in bile samples from patients with different prognoses. **(C)** Length distribution of eccDNAs in bile samples from patients with different prognoses. **(D)** KEGG pathway analysis of the upregulated eccDNAs in bile samples from patients with a poor prognosis. **(E)** KEGG pathway analysis of the upregulated eccDNAs in bile samples from patients with a better prognosis. KEGG, Kyoto Encyclopedia of Genes and Genomes.

We identified 2,195 eccDNAs exhibiting differential expression patterns according to the screening criteria (*p*-value <0.05 and |LogFC| > 1). As shown in Figure 3A, compared to group A, there were 1,602 upregulated eccDNAs and 593 downregulated eccDNAs in group B. A hierarchical clustering approach was employed to confirm the consistency of differently expressed eccDNAs in group B (n = 4) and group A (n = 5) (Figure 3B). Most of these candidate eccDNAs were observed either in group A or group B. The distribution in terms of length indicated comparable peak positions and ranges of eccDNAs among patients in distinct groups (Figure 3C). We conducted a GO analysis to investigate the functionalities of the genes linked to the differentially expressed eccDNAs. Regarding genes associated with upregulated eccDNAs in group B, the dominant biological process was cell adhesion, the main molecular function was catalytic activity, and the main cellular component was cell junction (Supplementary Material S2). Regarding genes associated with upregulated eccDNAs in group A, the dominant biological process was the modulation of chemical synaptic transmission, the main molecular function was cytoskeletal protein binding, and the main cellular component was cell protection (Supplementary Material S3). KEGG pathway analysis revealed that the genes associated with the upregulated eccDNAs in group B are involved in sphingolipid signaling, apelin signing, and tumor-related signaling pathways, such as the MAPK and WNT signaling pathways (Figure 3D). Genes associated with the upregulated eccDNAs in group A were involved in circadian entrainment and adherens junctions (Figure 3E).

### 3.5 Identification of the differentially expressed eccDNAs in the plasma between group A and group B

**FIGURE 4:**
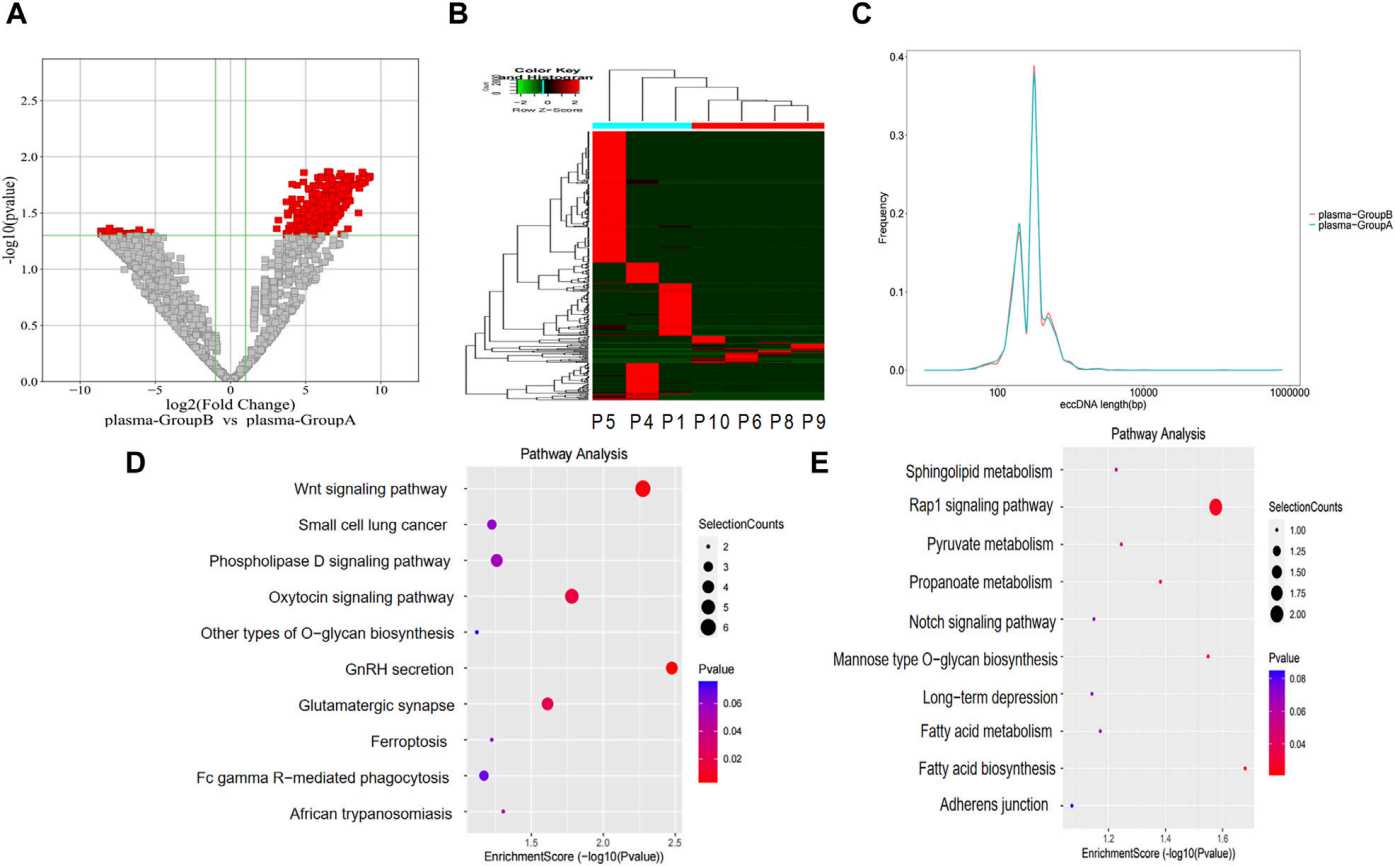
Differentially expressed eccDNAs in plasma between group A and group B. **(A,B)** Scatter and cluster plots showing the differentially expressed eccDNAs in plasma samples from patients with different prognoses. **(C)** Length distribution of eccDNAs in plasma samples from patients with different prognoses. **(D)** KEGG pathway analysis of the upregulated eccDNAs in the plasma samples from patients with a poor prognosis. **(E)** KEGG pathway analysis of the upregulated eccDNAs in the plasma samples from patients with a better prognosis.

The level of eccDNA expression in plasma samples in groups A and B was compared as described for the bile samples. Compared with group A, there were 358 upregulated eccDNAs and 41 downregulated eccDNAs in group B (Figures 4A, B). The length distribution also showed similar peak locations and length spans of eccDNAs in the different groups (Figure 4C). GO analysis indicated that regarding genes associated with upregulated eccDNAs in group B, the dominant biological process was the regulation of postsynaptic membrane neurotransmitter receptor levels, the main molecular function was transmembrane receptor protein phosphatase activity, and the main cellular component was the cytoplasm (Supplementary Material S4). Regarding genes associated with upregulated eccDNAs in group A, the dominant biological process was protein homotetramerization, the main molecular function was amino acid transmembrane transporter activity, and the main cellular component was nuclear matrix (Supplementary Material S5). KEGG pathway analysis revealed that the genes associated with the upregulated eccDNAs in group B are involved in gonadotropin-releasing hormone (GnRH) secretion and the WNT signaling pathway (Figure 4D). Genes associated with the upregulated eccDNAs in group A are involved in the Ras-related protein 1 (RAP1) signaling pathway (Figure 4E).

### 3.6 Construction of an eccDNA-related gene prognostic model for cholangiocarcinoma

**FIGURE 5:**
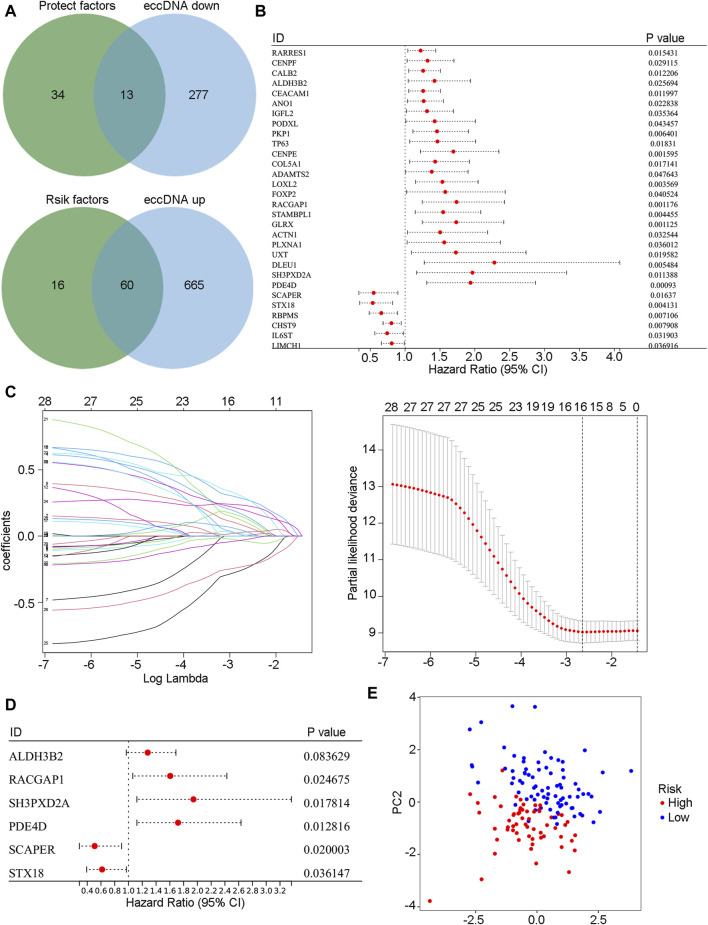
The prognosis model was constructed based on eccDNA-related genes. **(A)** The Venn diagram shows the intersection of risk genes with highly expressed eccDNA genes in the bile of group B patients and the intersection of protective genes with low-expressed eccDNA genes in the bile of group B patients. **(B)** Univariate Cox regression results of intersecting genes in the merged cohort (*p*-value <0.05). **(C)** The intersecting genes were further analyzed by LASSO-Cox regression analysis. **(D)** Model genes were selected by stepwise regression multivariate Cox regression analysis. **(E)** Model genes were used to conduct a PCA of the merged cohort. PCA, principal component analysis.

First, the difference in annotated eccDNA genes in the bile of patients in group B and group A was screened. Then, the merged cohort (n = 142) was randomly divided into a training cohort (n = 102) and a validation cohort (n = 40) at a 7:3 ratio. Univariate Cox regression analysis was performed on differentially expressed eccDNA-related genes in the training cohort to screen out genes related to cholangiocarcinoma patient prognosis. Among these differentially expressed eccDNA-related genes, those with risk coefficients >1 were regarded as risk genes, and those with risk coefficients <1 were considered protective genes. The risk gene overlap with the upregulated eccDNA-related genes in the bile of patients with poor prognosis after HAIC was assessed, and a total of 13 genes were obtained. After overlapping the protective genes with the upregulated eccDNA-related genes in the bile of patients with better prognosis, 60 genes were obtained (Figure 5A). Figure 5B shows the univariate Cox analysis results of the 73 eccDNA genes (*p*-value <0.05). LASSO Cox regression analysis was further used in the training cohort, a 10-fold cross-validation method was adopted, and 16 genes were identified according to the optimal λ value (Figure 5C). Finally, multivariate Cox regression analysis and stepwise regression methods were used to construct the prognostic model. The model genes ALDH3B2, RACGAP1, SH3PXD2A, PDE4D, SCAPER and STX18 were obtained. Each patient’s risk score was calculated according to the multivariate Cox regression risk coefficient and gene expression values as follows =0.245*ALDH3B2+0.473*RACGAP1+0.664*SH3PXD2A+0.541*PDE4D−0.677*SCAPER−0.483*STX18 . The patients were divided into high-risk and low-risk groups according to the median risk score (Figure 5D). Principal component analysis (PCA) showed that this model could better classify patients into these two groups (Figure 5E).

### 3.7 Identification of the risk characteristics and the predictive power of the model

**FIGURE 6:**
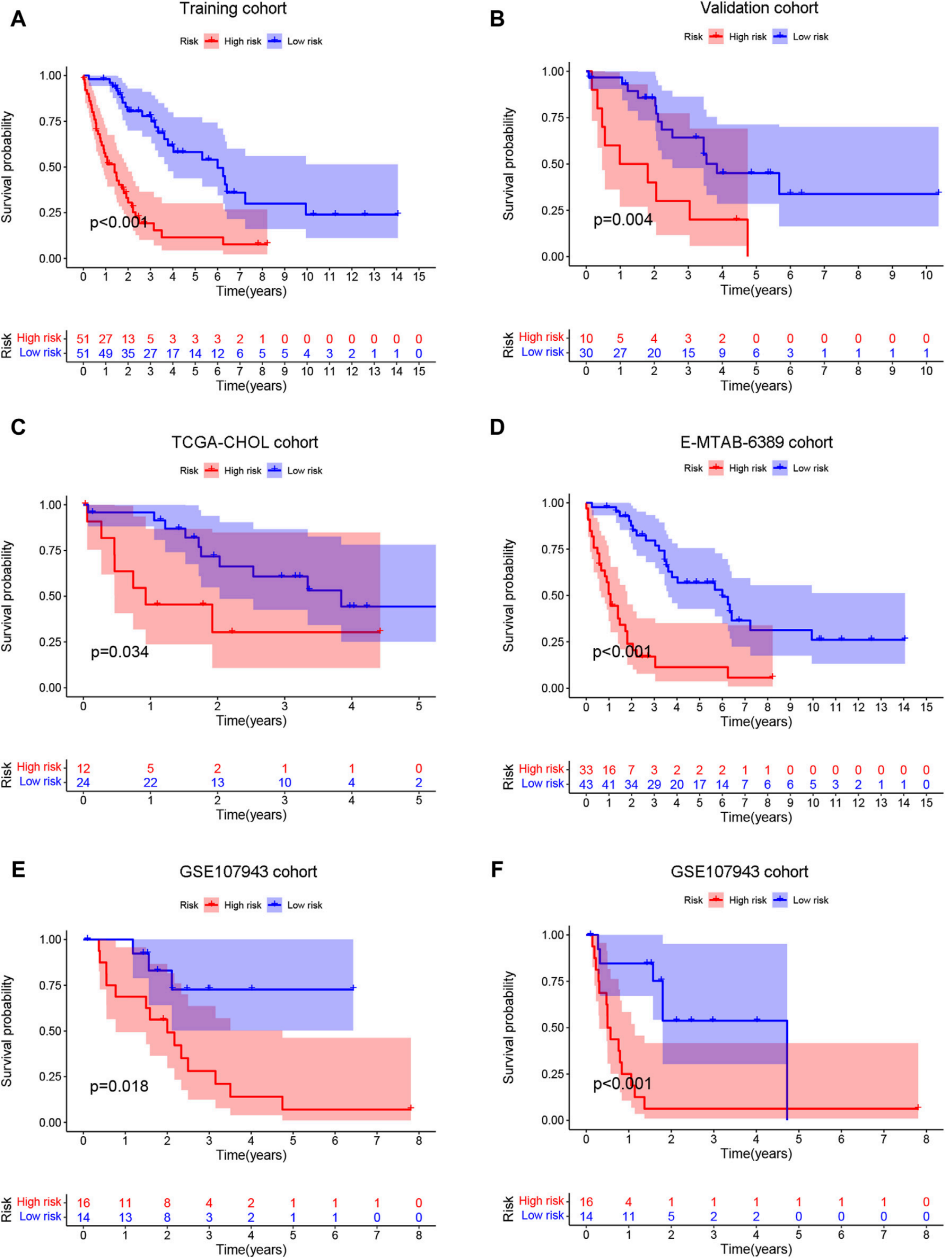
Survival prediction ability of prognostic models. The patients were categorized into high- and low-risk groups according to the risk score median. Then, the training **(A)**, validation **(B)**, TCGA-CHOL **(C)**, E-MTAB-6389 **(D)**, and GSE107943 **(E)** cohorts were employed to generate overall survival K‒M survival curves. **(F)** The GSE107943 cohort was also utilized to construct a disease-free survival K‒M curve.

**FIGURE 7:**
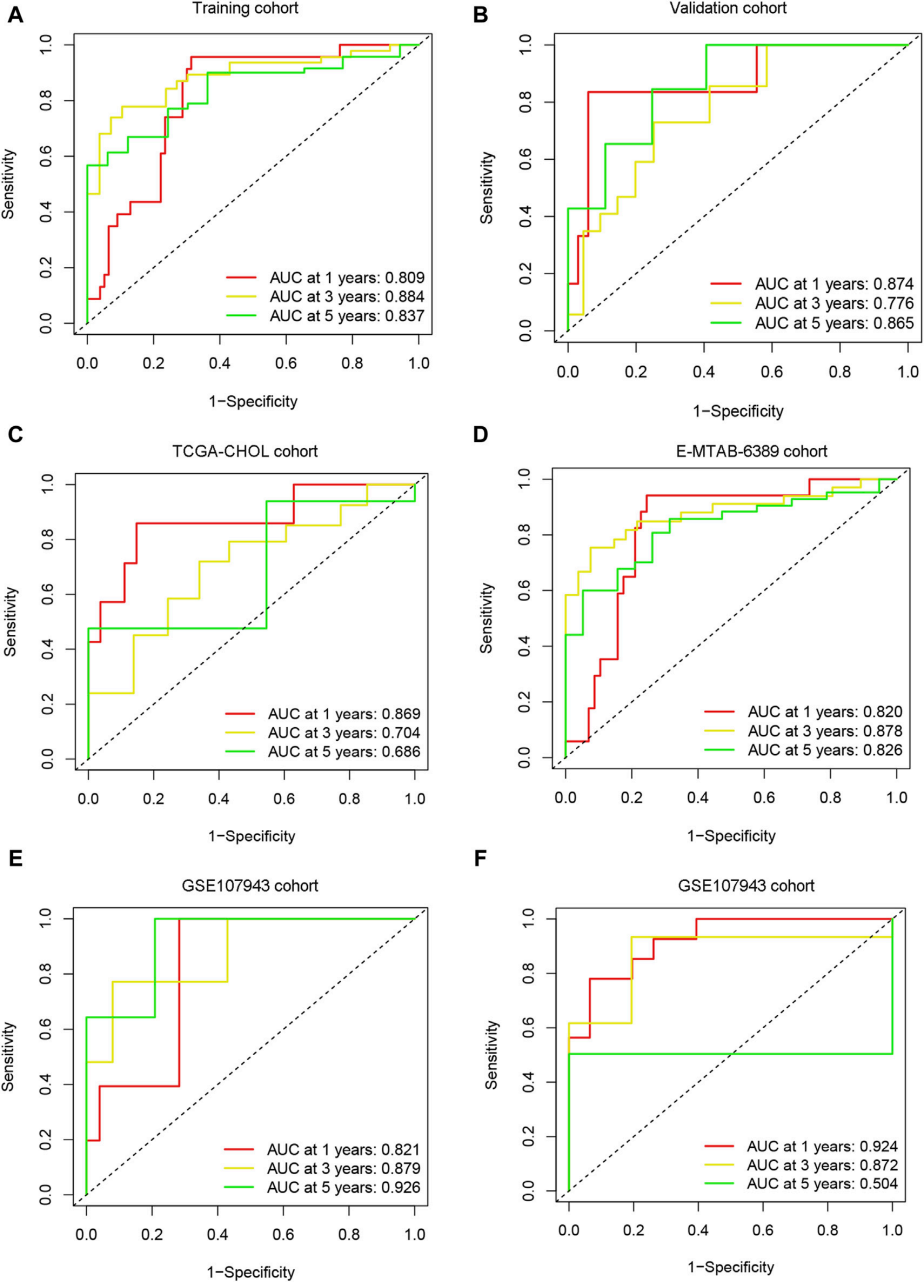
Model predictive performance. ROC analysis was performed on the risk scores in the training **(A)**, validation **(B)**, TCGA-CHOL **(C)**, E-MTAB-6389 **(D)**, and GSE107943 **(E)** cohorts, and the AUC value was calculated for 1-, 3-, and 5-year overall survival. **(F)** ROC analysis was performed for risk scores using the GSE107943 cohort, and AUC values were calculated for disease-free survival at 1, 3, and 5 years.

In the training (n = 102) (Figure 6A), validation (n = 40) (Figure 6B), TCGA-CHOL (n = 36) (Figure 6C), E-MTAB-6389 (n = 76) (Figure 6D), and GEO107943 (n = 30) (Figure 6E) cohorts, the OS prognosis in low-risk patients was significantly better than that in high-risk patients. Furthermore, the low-risk group demonstrated better disease-free survival (DFS) outcomes than the high-risk group (Figure 6F). ROC curves were plotted for OS and DFS at 1-, 3-, and 5-year intervals to assess the predictive capability of the model, revealing a high level of diagnostic efficacy. In the training cohort, the AUC for overall survival was 0.809 at 1 year, 0.884 at 3 years, and 0.837 at 5 years (Figure 7A). The validation cohort displayed AUC values of 0.874, 0.776, and 0.865 for 1-, 3-, and 5-year intervals, respectively (Figure 7B). The AUCs for the OS outcomes in the TCGA-CHOL, GSE107943, and E-MTAB-6389 datasets are shown in Figures 7C–E. Within the GSE107943 cohort, the AUC values for disease-free survival at 1, 3, and 5 years were 0.924, 0.872, and 0.504, respectively (Figure 7F). The lower AUC value for 5-year DFS is due to the limited number of patients with DFS outcomes exceeding 5 years. Overall, we demonstrated that the model could predict cholangiocarcinoma patient OS and DFS outcomes.

### 3.8 Immune characteristics of the prognostic model

**FIGURE 8:**
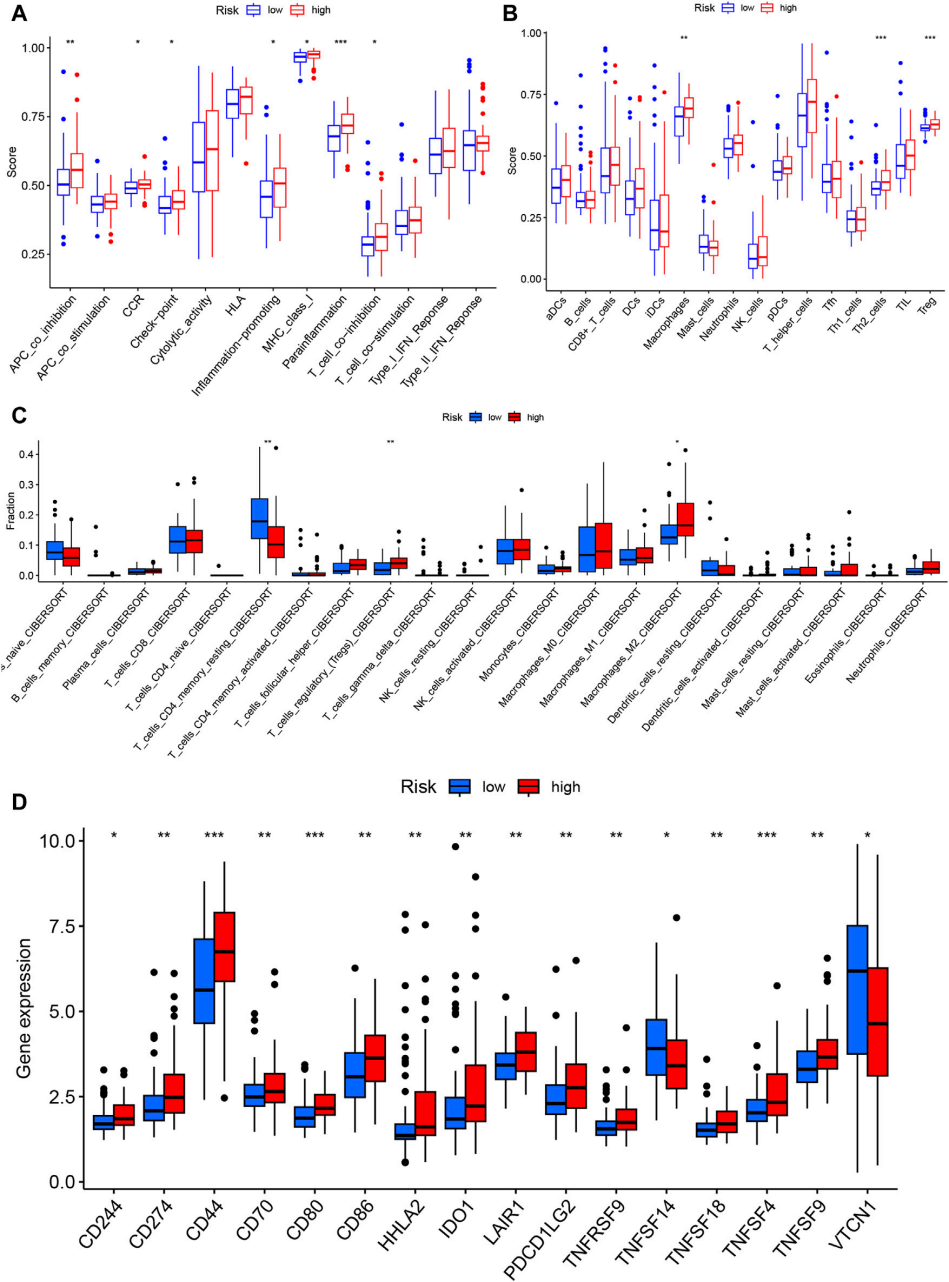
Immune characteristics of the prognostic model. Single-sample gene set analysis (ssGSEA) was used to calculate immune function and immune cell gene set enrichment scores for each sample to determine differential immune function **(A)** and immune cell infiltration **(B)** in the high- and low-risk groups. **(C)** The CIBERSORT algorithm was used to evaluate differential immune cell infiltration between the high- and low-risk groups. **(D)** Differential expression of immune checkpoint genes between the high- and low-risk groups.

**FIGURE 9:**
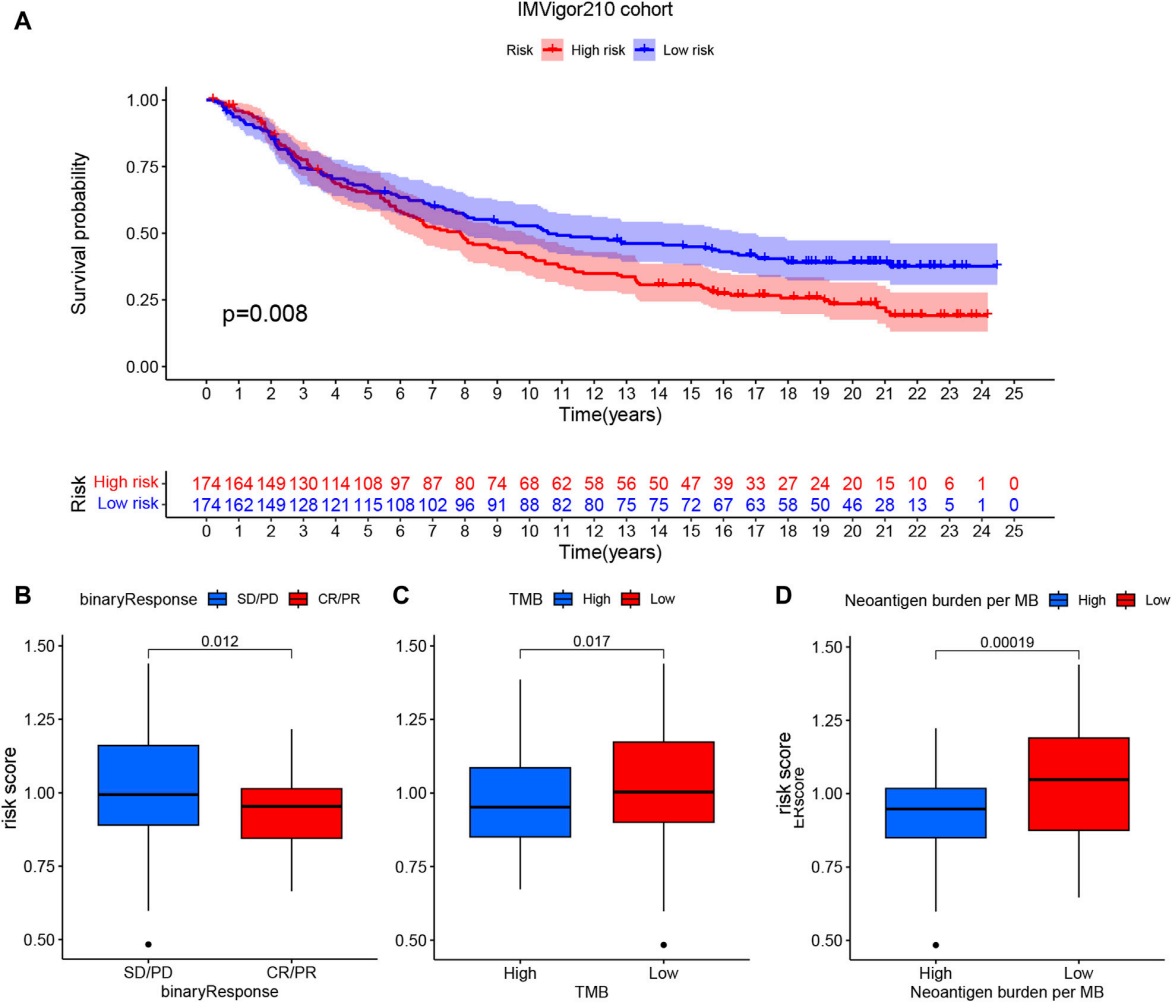
Potential impact of prognostic models on PD-L1 immunotherapy. **(A)** Survival curve indicating prognostic differences among patients categorized into high- and low-risk groups in the cohort receiving anti-PD-L1 treatment. **(B)** Connection between risk score and response to anti-PD-L1 treatment, **(C)** tumor mutational burden and **(D)** neoantigen TMB.

The ssGSEA algorithm was used to score the immune function and infiltration of immune cells in the high-risk and low-risk groups. The enrichment scores of APC coinhibition, immune checkpoint, and T-cell coinhibition were higher in the high-risk group (Figure 8A). Furthermore, the high-risk group showed greater infiltration of macrophages, Th2 cells, and Treg cells (Figure 8B). The CIBERSORT algorithm was also employed to analyze immune cell infiltration, and the high-risk group demonstrated greater Treg cell and M2-type macrophage infiltration (Figure 8C). Additionally, the expression of 29 immune checkpoints was examined in different risk populations: CD244, CD274, CD44, CD80, CD80, CD86, HHLA2, IDO1, LAIR1, PDCD1LG2, TNFRSF9, TNFSF14, TNFSF18, TNFSF4, CD244, CD274, CD44, CD80, CD86, HHLA2, IDO1, PDCD1LG2, TNFRSF9, TNFSF14, TNFSF18, and TNFSF4. TNFSF9 was highly expressed in the high-risk group, whereas VTCN1 was highly expressed in the low-risk group (Figure 8D). These findings indicate that the tumor microenvironment in the high-risk group is more immunosuppressive. An analysis was conducted to investigate the predictive potential of the model in immunotherapy using the IMVigor210 dataset, which comprises patients with bladder cancer undergoing anti-PD-L1 treatment. A model incorporating six specific eccDNA genes was established using this cohort. The findings indicated that individuals in the low-risk category who underwent anti-PD-L1 treatment exhibited a better prognosis (Figure 9A), and those who attained CR/PR following anti-PD-L1 therapy displayed reduced risk scores (Figure 9B). There was a positive correlation between the TMB and neoantigen TMB with the patient’s risk score, as demonstrated in Figures 9C, D. Based on these findings, it can be inferred that the 6-gene eccDNA model distinguishes disparate immune microenvironments in high-risk versus low-risk groups. Additionally, this model could provide valuable insights guiding the administration of immune checkpoint inhibitors in treatment approaches.

### 3.9 Functional enrichment analysis of the high-risk and low-risk groups

**FIGURE 10:**
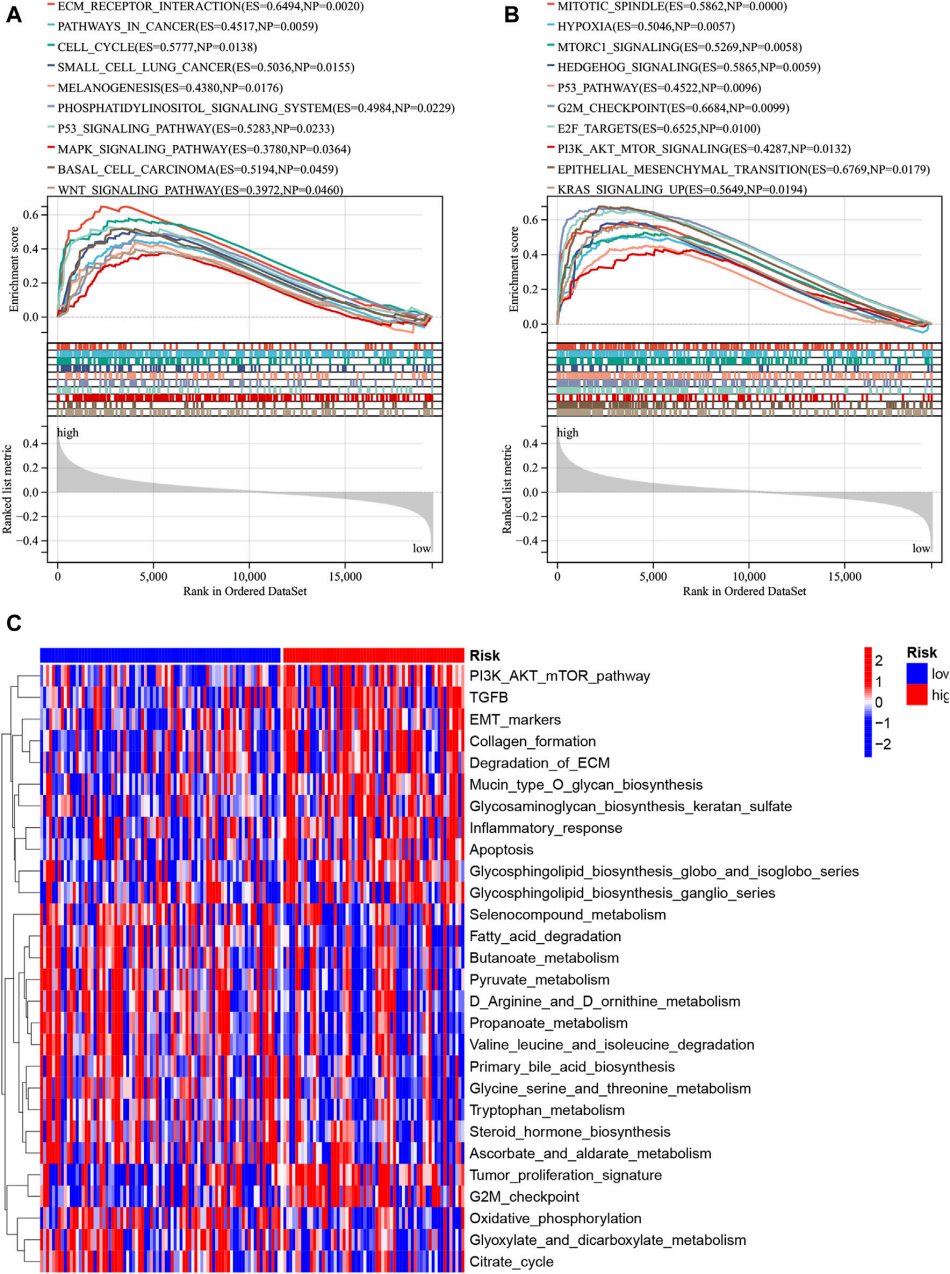
Functional enrichment disparities among the high- and low-risk groups were assessed. The enrichment analysis for the KEGG gene set **(A)** and hallmark gene set **(B)** was conducted via GSEA. Moreover, the disparity in enriched pathways between the high- and low-risk groups was examined utilizing GSVA **(C)**.

In the merged cohort, GSEA was employed to identify differing molecular functions and pathways between the high- and low-risk groups using the KEGG signaling pathway and hallmark gene set. The high-risk group displayed increased enrichment in numerous molecular pathways associated with cancer, including cancer pathway, small cell lung cancer, and Wnt signaling pathway (Figure 10A). Moreover, the high-risk group exhibited a high enrichment of gene sets linked to tumor cell proliferation (HALLMARK_MITOTIC_SPINDLE and HALLMARK_G2M_CHECKPOINT) and metastasis (HALLMARK_EPITHELIAL_MESENCHYMAL_TRANSITION), as indicated in Figure 10B. Gene set variation analysis (GSVA) was also employed to assess the degree of enrichment of cancer-related pathways in each sample and analyze pathway differences. The results demonstrated a significant increase in the enrichment of oncogene sets (Tumor_proliferation_signature, PI3K_AKT_mTOR_pathway and EMT_markers) in the high-risk group (Figure 10C). Collectively, these findings suggest that high-risk patients show an enrichment of multiple cancer-promoting pathways that contribute to a poor prognosis.

### 3.10 The characteristics of ALDH3B2, RACGAP1, SH3PXD2A, PDE4D, SCAPER, and STX18 in cholangiocarcinoma

**FIGURE 11:**
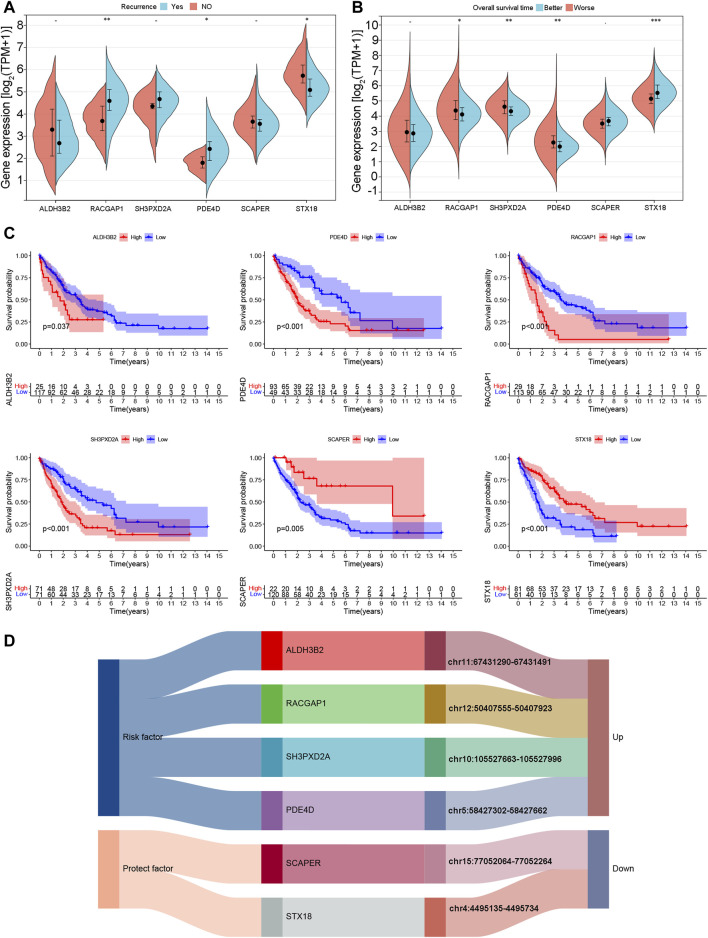
Expression and prognostic characteristics of model genes. **(A)** Expression of model genes in samples with and without recurrent disease. **(B)** Expression of model genes in better and poor overall survival outcome groups. **(C)** K‒M curves of model genes according to overall survival outcomes. **(D)** Correspondence between model genes and eccDNA locations.

The expression of ALDH3B2, RACGAP1, SH3PXD2A, PDE4D, SCAPER, and STX18 was analyzed in the TCGA-CHOL cohort. Samples from patients who experienced recurrence exhibited elevated expression of the risk genes RACGAP1 and PDE4D and reduced expression of the protective gene STX18 (Figure 11A). Patients were categorized into two groups according to the median OS duration: the better prognosis and poor prognosis groups. High expression of the risk genes PDE4D, RACGAP1, and SH3PXD2A was observed in the poor prognosis group, whereas the better prognosis group exhibited high expression of the protective gene STX18 (Figure 11B). The K‒M curves of the six genes were plotted for the merged cohort. The analysis revealed that higher expression levels of risk genes, such as ALDH3B2, PDE4D, RACGAP1, and SH3PXD2A, were associated with a worse patient prognosis. Lower expression levels of the protective genes SCAPER and STX18 were also linked to a worse patient prognosis (Figure 11C). Figure 11D shows the correlation between the six eccDNA genes and eccDNA locations. The eccDNAs corresponding to four risk genes were highly expressed in the poor prognosis group (group B), while the eccDNAs corresponding to two protective genes were expressed at low levels in group B.

## 4 Discussion

In clinical practice, performing a core needle biopsy to obtain pCCA tissues is challenging because of its particular perihilar anatomical site and periductal infiltration growth pattern along the bile duct wall. There is a clinical need for reliable circulating biomarkers to predict treatment response and survival outcomes for pCCA patients. EccDNA has higher stability than linear DNA because it is circular, extrachromosomal, and exonuclease-resistant (Gaubatz and Flores, 1990). The present study first showed that eccDNA can be successfully detected in the bile and plasma of pCCA patients, with more eccDNAs detected in the bile than in the plasma. Almost all eccDNAs detected in the plasma could also be detected in the bile, possibly due to the special location and characteristics of pCCA. Moreover, there was good consistency in the length and chromosome distribution features between eccDNAs in bile and matched plasma samples.

This study examined the prognostic significance of differentially expressed eccDNAs in pCCA patients. The investigation revealed a strong correlation between the expression of eccDNAs and patient prognosis. Furthermore, an analysis of genes associated with the increased levels of eccDNAs in patients with poor survival demonstrated enrichment in the MAPK pathway (in bile samples) and WNT pathway (in both bile and plasma samples). These two pathways have been extensively implicated in the progression of cancer and are known to contribute to unfavorable clinical outcomes (Taciak et al., 2018; Kciuk et al., 2022). The upregulated eccDNAs enriched in the MAPK pathway in patients with poor survival outcomes originated from cancer-related genes, such as AKT3 and MAPK. Several studies have elucidated AKT3-specific oncogenic roles (Cristiano et al., 2006; Grabinski et al., 2014; Madhunapantula and Robertson, 2017). The eccDNA located on chr19:3018721-3019021, originating from the TLE-2 gene, was upregulated and enriched in the WNT pathway in both bile and plasma samples from patients with poor survival outcomes. This finding indicates the value of TLE-2 in eccDNAs for predicting poor survival outcomes. However, to date, few studies have investigated the relationship between the expression of TLE and the development of solid tumors (Dayyani et al., 2008; Ramasamy et al., 2016). The detailed function of the MAPK and WNT pathways in pCCA and eccDNA enrichment in these pathways requires further exploration to determine whether they are prognosis-related or efficacy-related factors for pCCA patients.

A prognostic model (including ALDH3B2, RACGAP1, SH3PXD2A, PDE4D, SCAPER and STX18) was constructed for cholangiocarcinoma patients according to the differentially expressed eccDNA-related genes in the bile to further analyze the function of eccDNA annotated genes and the regulation of cholangiocarcinoma. The model showed a relatively accurate ability to predict survival in public datasets and could distinguish cholangiocarcinoma patients’ different immune microenvironments. This prognostic model was consistent with previous studies on these genes in hepatobiliary malignancies. ALDH3B2 expression was significantly upregulated in perihilar cholangiocarcinoma tissues, which was also correlated with tumor stage in pCCA patients. ALDH3B2 promotes the proliferation and metastasis of cholangiocarcinoma by regulating ITGB1 expression (Wang et al., 2021). RACGAP1 is a suppressor gene in hepatocellular carcinoma (HCC), and its expression can predict early HCC recurrence. In addition, silencing RACGAP1 can inhibit HCC cell migration and invasion (Wang, Ooi, and Hui, 2011). PDE4D overexpression increases the dephosphorylation and activity of YAP, promoting the growth of hepatocellular carcinoma cells *in vitro* and *in vivo* (Ren et al., 2022). SH3PXD2A and STX18 promote or inhibit the development of other cancers, consistent with our prognostic model. SH3PXD2A forms a complex with ULK1 and MTOR, and the ULK1-SH3PXD2a-MMP14 axis upregulates the aggressiveness of ovarian cancer (Lin et al., 2023). Downregulation of STX18 can significantly enhance the growth of MCF-7 breast cancer cells (Bassett et al., 2008). SCAPER can bind cyclin A/Cdk2 in the endoplasmic reticulum. Overexpression of SCAPER transports cyclin A of the nucleus, delays G2/M phase transition, and prevents cyclin A from interacting with Cdk (Tsang and Dynlacht, 2008).

As eccDNA was more abundant in bile than in peripheral blood, eccDNA-related genes in bile were applied to construct the prognostic model. Moreover, as mentioned above, previous studies have revealed that the six genes included in the prognostic model can promote or inhibit the progression of cholangiocarcinoma (or other cancers), confirming that these genes play a role in cancer progression and prognosis. In addition, GSEA enrichment analysis based on the prognostic model showed that several cancer-promoting molecular pathways were more enriched in the high-risk group, including the MAPK and WNT signaling pathways, consistent with the KEGG enrichment analysis of upregulated bile eccDNAs in pCCA patients with worse survival outcomes. These results indicate that the model based on bile eccDNA-related genes is reliable, and bile eccDNAs might serve as important survival biomarkers for pCCA patients.

Our research has several limitations. First, because this was a single-center study based on several bile and plasma samples, the results require further validation in a large cohort of pCCA patients. Second, the high-throughput sequencing results were not validated by traditional PCR or Sanger sequencing. Finally, although we found enrichment of eccDNA-associated genes in the MAPK and WNT pathways, we did not further explore their molecular mechanisms in pCCA.

## 5 Conclusion

In summary, this study demonstrates that eccDNAs can be detected in the bile and plasma of pCCA patients and demonstrates similar length distribution ranges and peak values. There were substantially more eccDNAs detected in the bile than in the plasma, and the eccDNAs detected in the bile overlapped and comprised all the eccDNAs detected in the plasma. The enrichment of eccDNAs in the WNT and MAPK pathways might be associated with a poor prognosis. In addition, the prognostic model of cholangiocarcinoma constructed using differentially expressed eccDNA-related genes can predict patient survival times, differentiate the immune microenvironment of different patients, and predict immunotherapy efficacy. The value of bile eccDNAs as potential liquid biomarkers of survival in pCCA patients requires further investigation.

## Data Availability

The data presented in the study are deposited in the Sequence Read Archive (SRA) repository, accession number: PRJNA1113366.

## Supplementary material

SUPPLEMENTARY MATERIAL S1 Length distribution of eccDNAs in the bile and matched plasma samples. Supplementary Figures S1–S6: Length distribution of eccDNAs in the bile and matched plasma samples of patients 1, 4, 6, 8, 9, and 10. eccDNAs, extrachromosomal circular DNAs.

SUPPLEMENTARY MATERIAL S2 GO analysis of bile eccDNAs. Cellular components, molecular functions, and biological processes associated with the upregulated eccDNAs in the bile of group B.

SUPPLEMENTARY MATERIAL S3 GO analysis of bile eccDNAs. Cellular components, molecular functions, and biological processes associated with the upregulated eccDNAs in the bile of group A.

SUPPLEMENTARY MATERIAL S4 GO analysis of plasma eccDNAs. Cellular components, molecular functions, and biological processes associated with the upregulated eccDNAs in the plasma of group B.

SUPPLEMENTARY MATERIAL S5 GO analysis of plasma eccDNAs. Cellular components, molecular functions, and biological processes associated with the upregulated eccDNAs in the plasma of group A.

SUPPLEMENTARY MATERIAL S6
**(A)** The UMAP shows that the samples within each data set are clustered together before batch effect removal, indicating a batch effect between different data sets. **(B)** After batch effect removal, the samples in each data set are mutually clustered, which is not limited to clustering within the respective data set, indicating that batch effect removal is optimal.

## Abbreviations

eccDNA, extrachromosomal circular DNAs; GO, gene ontology; HAIC, hepatic arterial infusion chemotherapy; KEGG, Kyoto Encyclopedia of Genes and Genomes; MAPK, mitogen-activated protein kinase; OS, overall survival; pCCA, perihilar cholangiocarcinoma; RAP1, Ras-related protein 1; GSEA, gene set enrichment analysis; GSVA, gene set variation analysis; PCA, principal component analysis; LASSO Cox, analysis, least absolute shrinkage, and selection operator Cox analysis; ssGSEA, single-sample gene set analysis.
