# Supplementary figures and images for: Extrachromosomal circular DNA (eccDNA) characteristics in the bile and plasma of advanced perihilar cholangiocarcinoma patients and the construction of an eccDNA-related gene prognosis model
Source: Front Cell Dev Biol. 2024 Jun 6;12:1379435. doi: 10.3389/fcell.2024.1379435 (PMC11187006; doi:10.3389/fcell.2024.1379435)

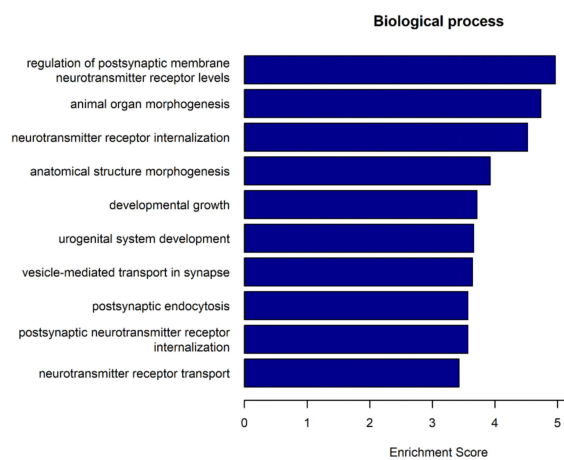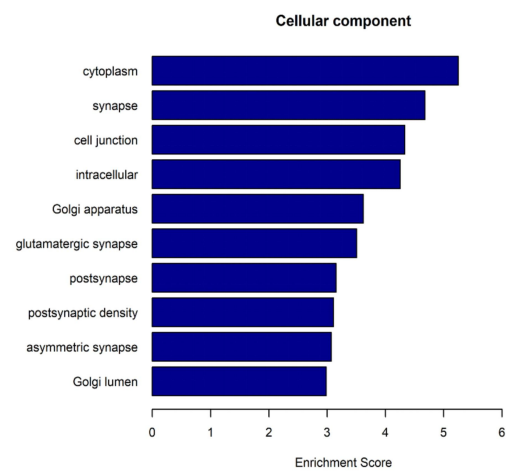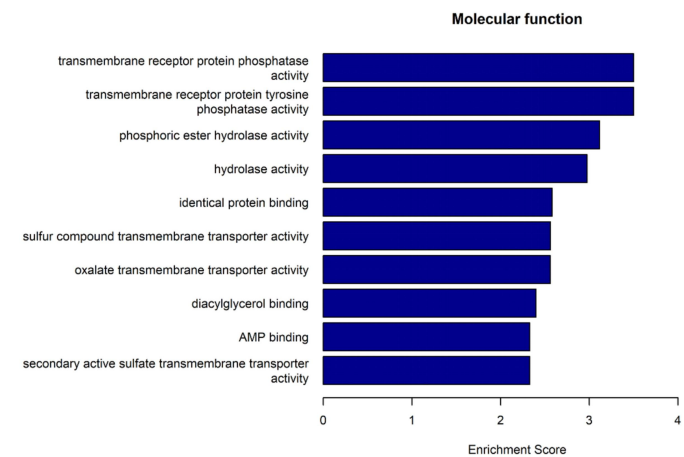

Supplement: Supplementary file 1 [file Image5.PDF]

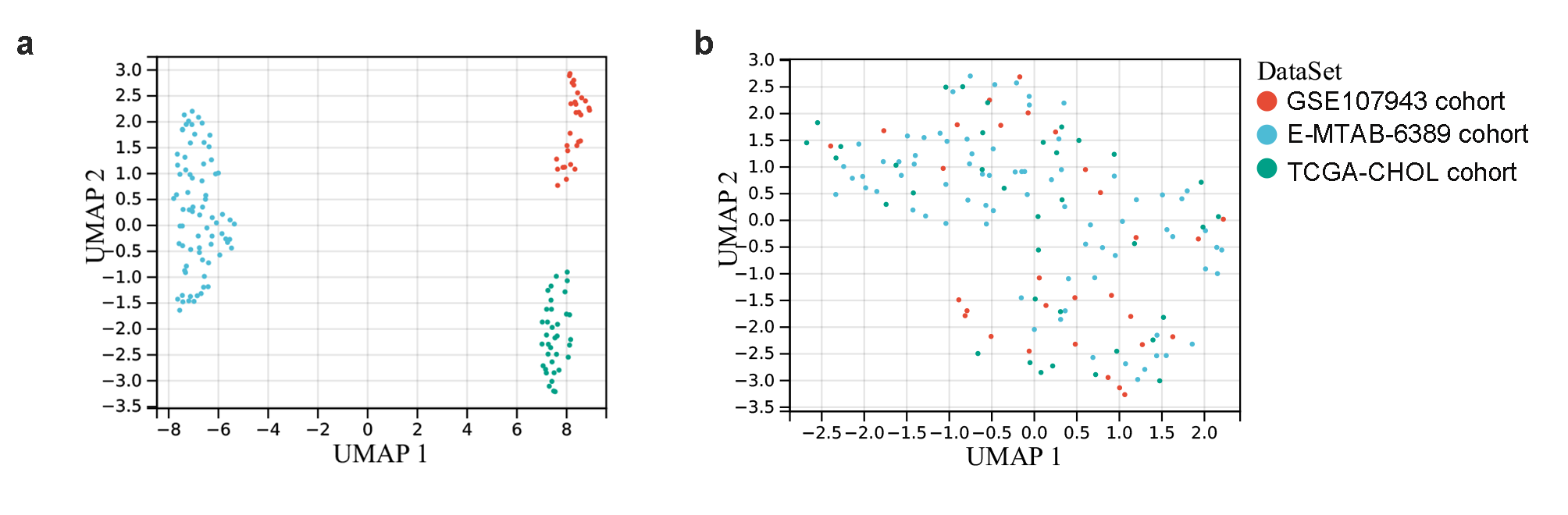

Supplement: Supplementary file 2 [file Image6.TIF]

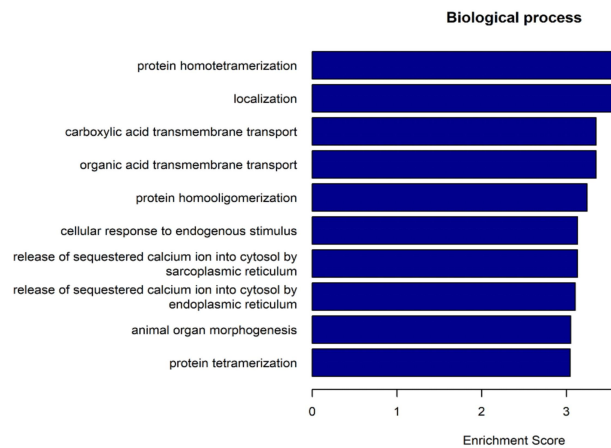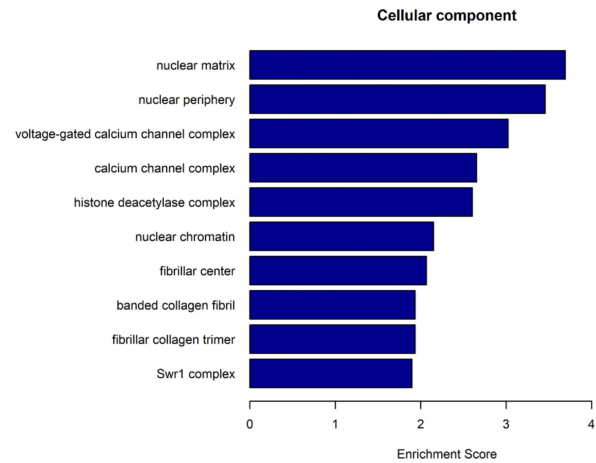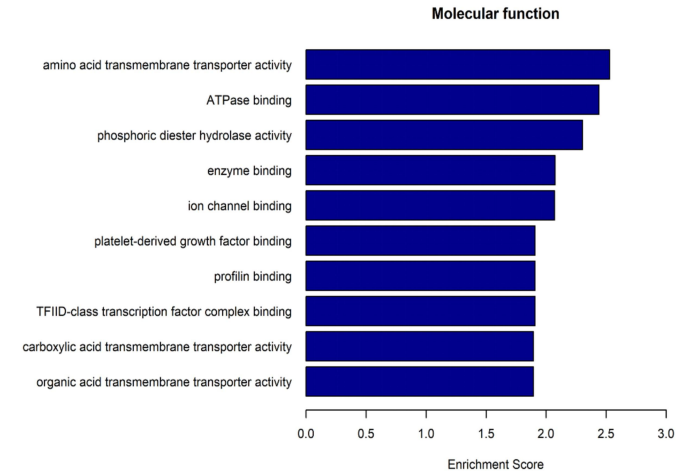

Supplement: Supplementary file 4 [file Image4.PDF]

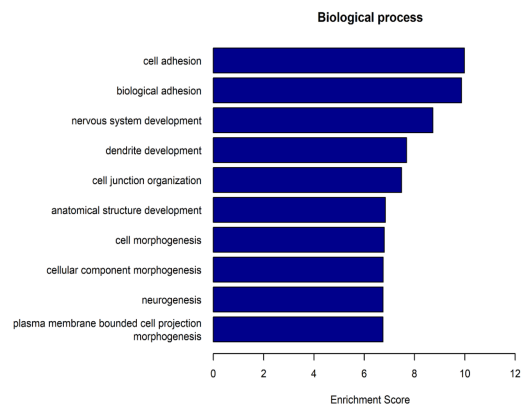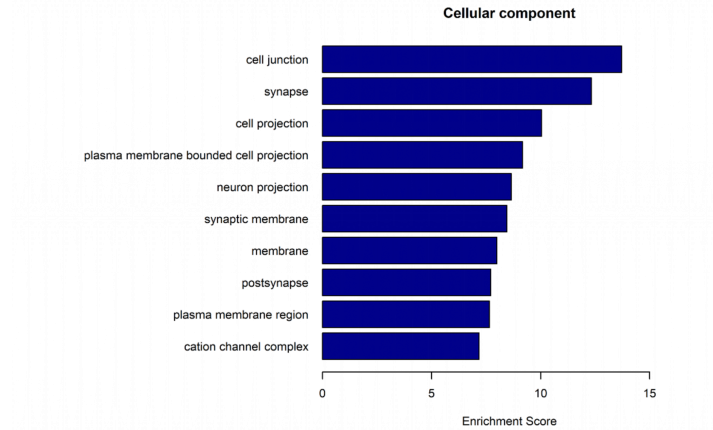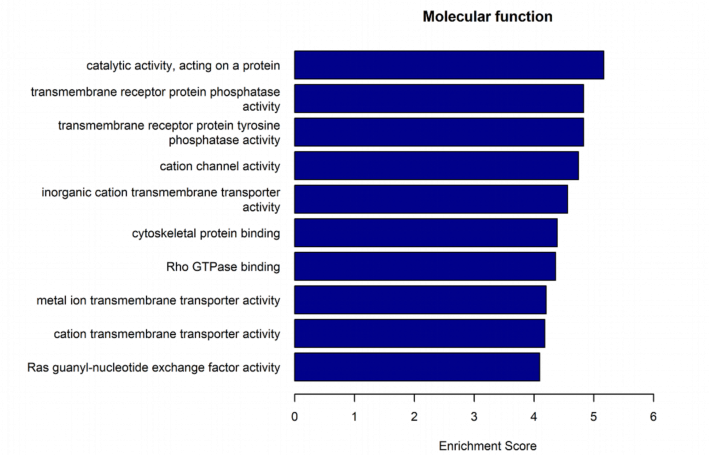

Supplement: Supplementary file 5 [file Image2.PDF]

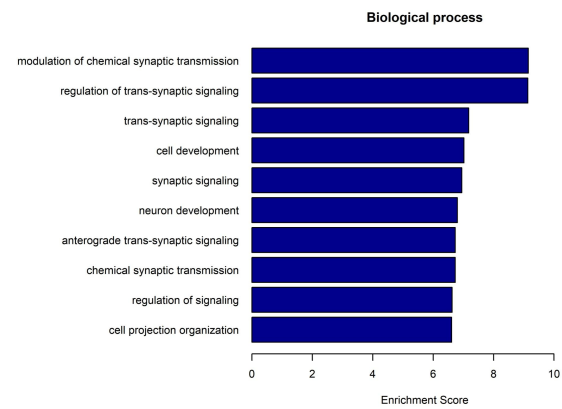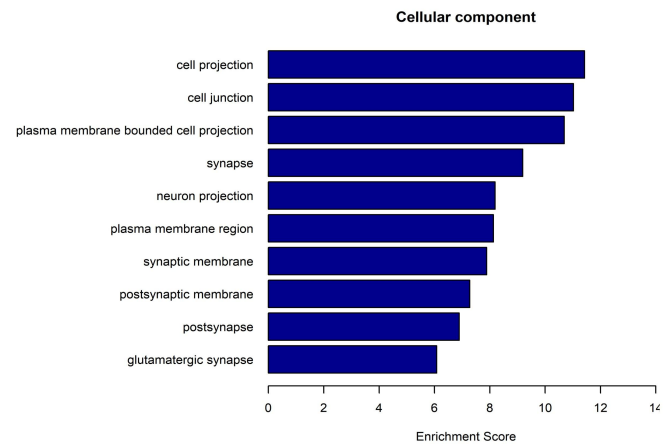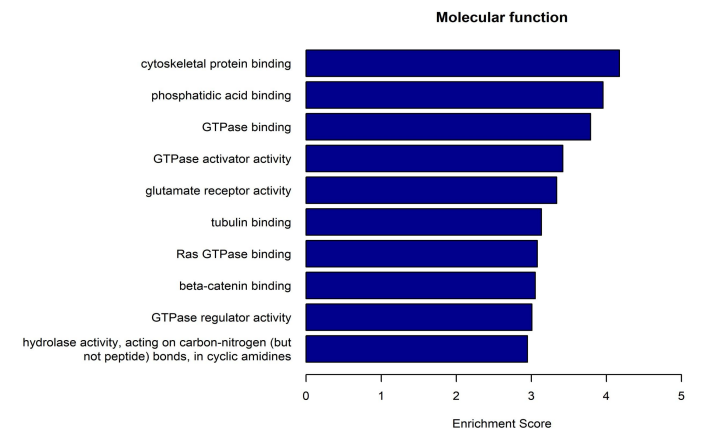

Supplement: Supplementary file 6 [file Image3.PDF]

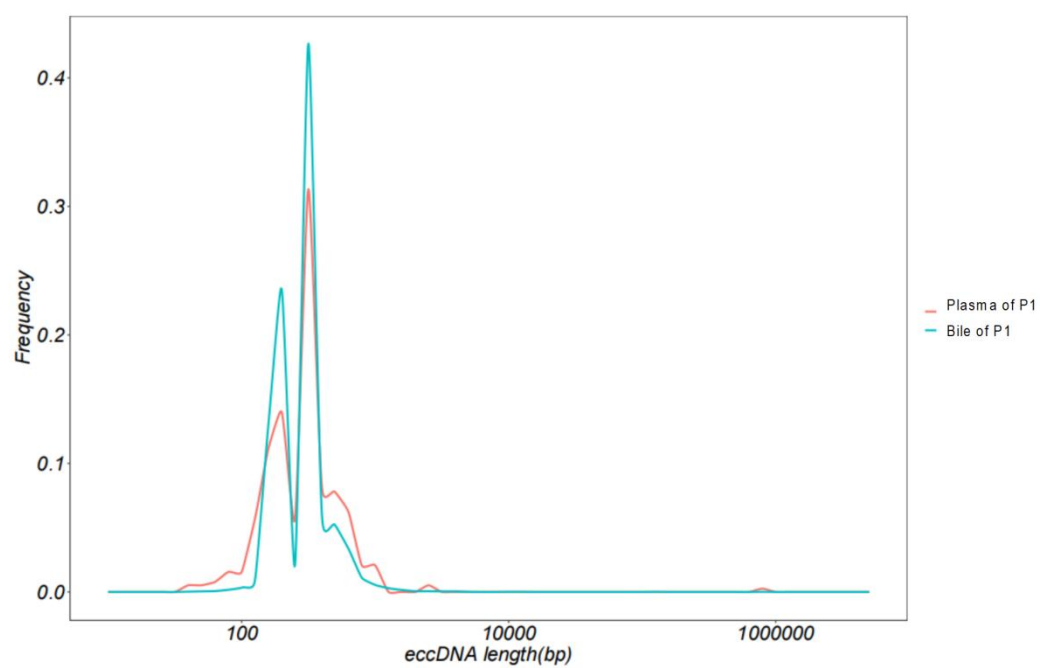

sFig 1

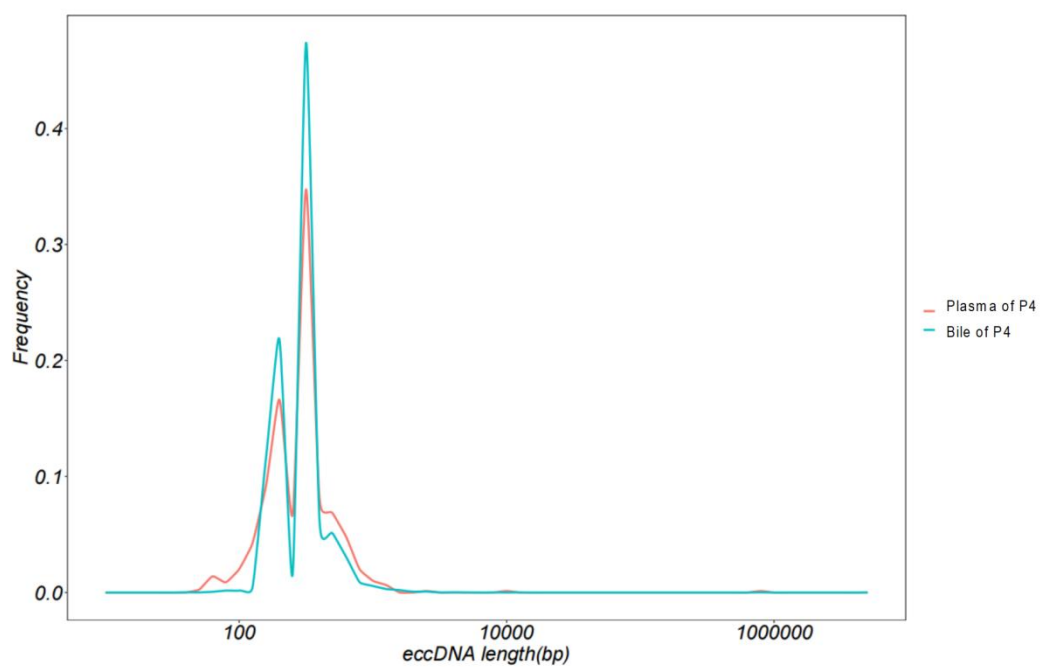

sFig 2

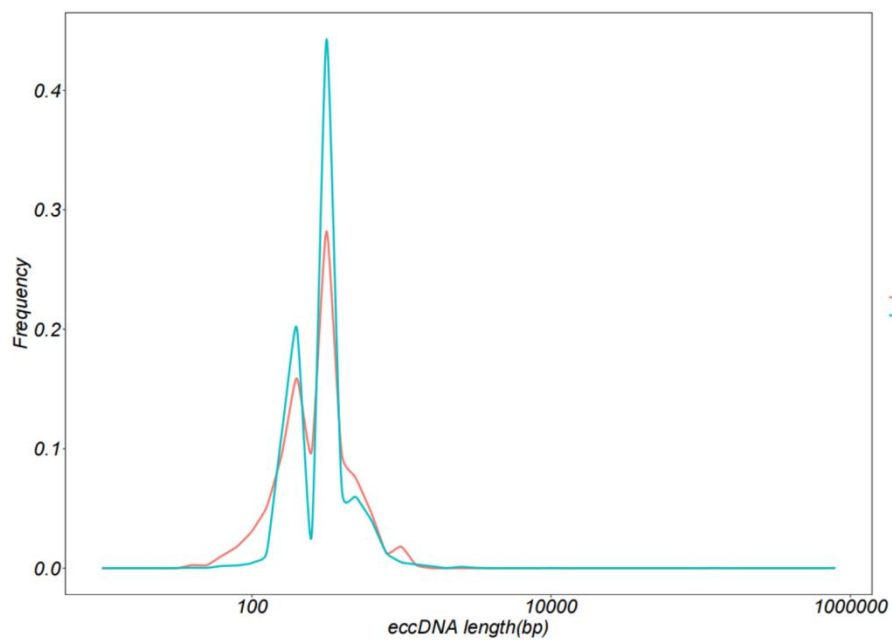

sFig 3

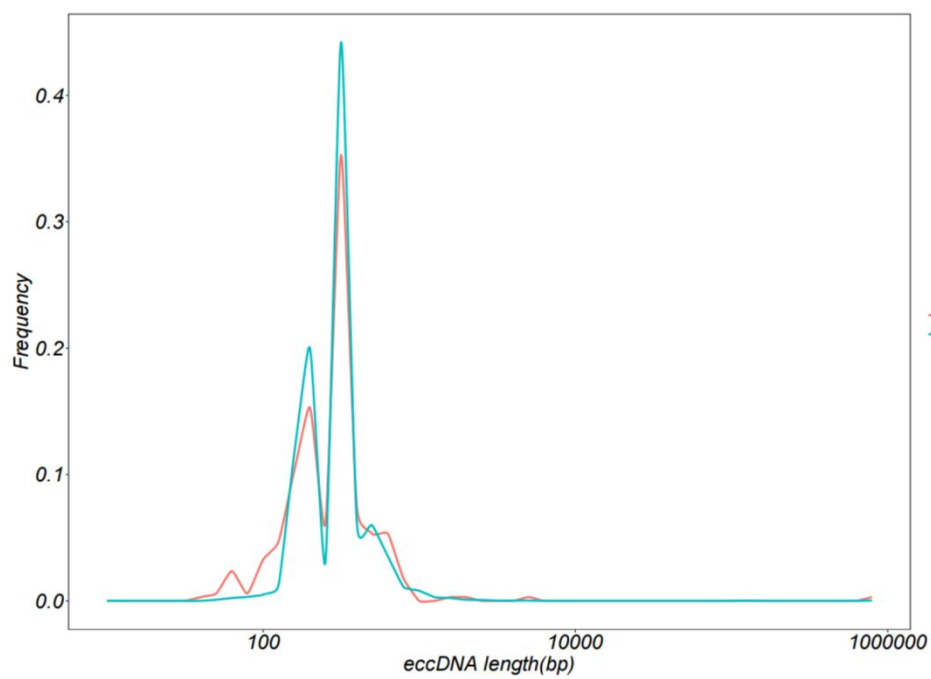

sFig 4

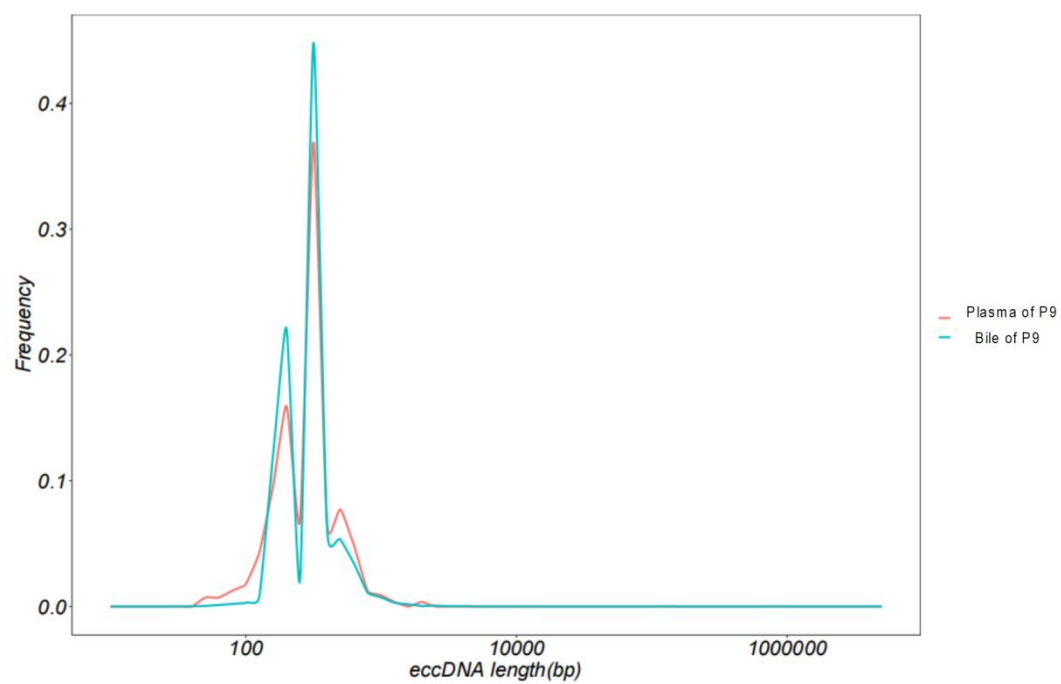

sFig 5

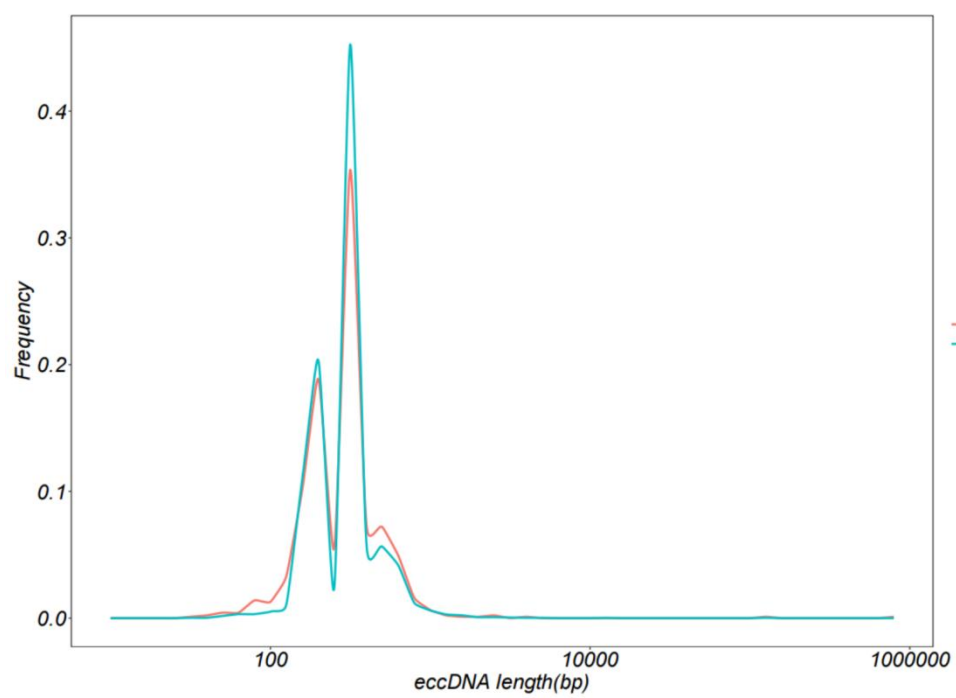

sFig 6

Supplement: Supplementary file 8 [file Image1.PDF]
