# Supplementary material for: Extrachromosomal circular DNA (eccDNA) characteristics in the bile and plasma of advanced perihilar cholangiocarcinoma patients and the construction of an eccDNA-related gene prognosis model
Source: Front Cell Dev Biol. 2024 Jun 6;12:1379435. doi: 10.3389/fcell.2024.1379435 (PMC11187006; doi:10.3389/fcell.2024.1379435)
Supplement: Supplementary file 3 [file Table1.DOCX]

**Table 1.** Summary of patient baseline characteristics

| Patient | P1 | P2 | P3 | P4 | P5 | P6 | P7 | P8 | P9 | P10 |
| --- | --- | --- | --- | --- | --- | --- | --- | --- | --- | --- |
| Age (Y) | 56 | 68 | 34 | 53 | 42 | 92 | 58 | 70 | 54 | 73 |
| Sex | Female | Male | Female | Female | Male | Male | Male | Male | Male | Male |
| HBV infection | None | Yes | None | None | None | None | None | Yes | None | None |
| Child-Pugh class | B | B | B | B | B | A | B | A | B | B |
| CEA (ng/mL) | 2.45 | 4.06 | 1.23 | 1.93 | 1.04 | 4.42 | 5.44 | 1.99 | 2.04 | 6.68 |
| CA19-9 (U/mL) | 1978 | 2614 | 2003 | 330 | 1508 | 94.94 | 246.3 | 1080 | 11.29 | 7401 |
| Total bilirubin (μmol/L) | 93.6 | 100 | 97.75 | 134.1 | 68.8 | 31.9 | 78.4 | 74 | 108.4 | 147.4 |
| ECOG performance status | 0 | 0 | 1 | 0 | 0 | 0 | 1 | 1 | 0 | 1 |
| Extent of disease | N0M0 | N1M0 | N2M1 | N0M0 | N1M0 | N0M0 | N0M0 | N0M0 | N0M0 | N0M0 |
| HAIC cycles | 2 | 2 | 2 | 2 | 2 | 4 | 6 | 6 | 6 | 6 |
| PFS (months) | 1.6 | 2.2 | 1.9 | 2.0 | 1.8 | 10.0 | 12.0 | 19.0 | 25.0 | 6.0+* |
| OS (months) | 5.5 | 3.0 | 2.5 | 6.0 | 2.0 | 25.0 | 14.0 | 25.0 | 30.5 | 22.5 |
| Bile sample | Yes | Yes | Yes | Yes | None | Yes | Yes | Yes | Yes | Yes |
| Plasma sample | Yes | None | None | Yes | Yes | Yes | None | Yes | Yes | Yes |
| Treatment response | PD | PD | PD | PD | PD | PR | PR | SD | PR | PR |
| Group | B | B | B | B | B | A | A | A | A | A |

*: Based on the date of last imaging, the PFS of P10 was recorded as 6+ months

CEA: carcinoembryonic antigen; CA19-9: carbohydrate antigen 19-9; HAIC: hepatic arterial infusion chemotherapy; PFS: progression-free survival; OS: overall survival; PR: partial response; SD: stable disease; PD: progressive disease; HBV: hepatitis B virus; ECOG, Eastern Cooperative Oncology Group
