# Supplementary material for: Extrachromosomal circular DNA (eccDNA) characteristics in the bile and plasma of advanced perihilar cholangiocarcinoma patients and the construction of an eccDNA-related gene prognosis model
Source: Front Cell Dev Biol. 2024 Jun 6;12:1379435. doi: 10.3389/fcell.2024.1379435 (PMC11187006; doi:10.3389/fcell.2024.1379435)
Supplement: Supplementary file 7 [file Table2.DOCX]

code data-working sheets：

https://www.jianguoyun.com/p/DeHhnPMQn5-tDBjhxbUFIAA
